# Imagine All the People: A Guided Internet-Based Imagery Training to Increase Assertiveness among University Students—Study Protocol for a Randomized Controlled Trial

**DOI:** 10.3390/healthcare11131874

**Published:** 2023-06-28

**Authors:** Micaela Di Consiglio, Jessica Burrai, Emanuela Mari, Anna Maria Giannini, Alessandro Couyoumdjian

**Affiliations:** Department of Psychology, Faculty of Medicine and Psychology, Sapienza University of Rome, 00185 Rome, Italy; micaela.diconsiglio@uniroma1.it (M.D.C.); jessica.burrai@uniroma1.it (J.B.); e.mari@uniroma1.it (E.M.); annamaria.giannini@uniroma1.it (A.M.G.)

**Keywords:** assertive training, assertiveness, imagination, imagery technique, web-based intervention, guided self-help intervention, university students, communication style, social skills, well-being

## Abstract

The importance of communication skills for well-being and self-realization is widely accepted. Despite that, research on assertiveness and assertiveness training has declined significantly in recent decades. Consequently, traditional training does not consider the most recent novel technologies used to spread psychological interventions. This study proposes the development of ComunicaBene: a guided Internet-based imagery intervention to promote assertiveness. Moreover, it describes the study protocol for a randomized control trial to investigate the intervention’s efficacy and acceptability. Participants will be randomly assigned to an experimental (ComunicaBene) or waitlist control condition. ComunicaBene consists of different online training modules corresponding to three phases: psychoeducation, imaginative exposure, and in vivo-exposure. Each module provides participants with theoretical and practical content about needs, emotions, communication style, and assertiveness. Moreover, during the program, every student is supervised by a Tutor. Participants in the control condition will be included in a waiting list. Primary and secondary outcomes will include changes in assertiveness, well-being, emotional awareness, worry, and rumination. Outcomes will be assessed at pre- and post-intervention, and via a 6-month follow-up. We expect that the results will support the efficacy of ComunicaBene as an innovative, scalable, affordable, and acceptable intervention to spread assertive training through the Internet and among a broad population.

## 1. Introduction

Assertiveness includes communicating one’s needs, thoughts, and feelings clearly and directly while listening and considering the ones of others. Therefore, assertive individuals stand up for themselves without dominating the other person or letting themselves become overwhelmed [1,2]. Assertiveness can be described as a continuum, with the two extreme poles—the passive and aggressive poles—representing the absence of assertiveness and being characterized by excessive agreeableness and excessive hostility, respectively [3].

Assertiveness is a relevant factor associated with mental health and psychological well-being [4,5], self-esteem [6,7], psychological empowerment [8], the satisfaction of one’s own needs [4], and improved interpersonal relationships [9]. On the other hand, unassertiveness is a transdiagnostic factor contributing to the onset and maintenance of clinical problems [10]. For example, individuals with social anxiety often adopt passive and avoidant behaviors [11,12], repressing high levels of anger and hostility [13]. Moreover, unassertiveness correlates with depressive symptoms [5,14], contributing to both the onset and the deterioration of depressive symptoms [15,16]. Lastly, assertiveness negatively correlates with psychological distress [17]. 

Assertiveness is a skill that can be learned or improved. Assertive training aims to help individuals to change their self-perception, increase assertiveness, and reduce anxiety-based inhibitions. These interventions can stand alone or be part of more comprehensive interventions and usually include a series of cognitive and behavioral techniques, such as the behavioral rehearsal, modeling, role-playing, exposure, and cognitive restructuring [3]. Many studies suggests the effectiveness of assertive training in reducing depressive and social anxiety symptoms [18,19,20], improving communication skills [10], self-esteem [6,21], and psychological well-being [22]. 

Despite the widely accepted importance of assertiveness for well-being and self-realization, most conceptual and experimental studies about assertiveness have been published between the 1970s and 1990s. In the past decades, assertiveness training has largely disappeared as a standalone treatment. This decline could reflect the fact that research priorities moved on to other interests, such as specific mental disorders, or that assertiveness training has been integrated into more comprehensive interventions (e.g., social skill training) [10]. The decreased attention given to assertiveness in recent decades contributed to the fact that traditional assertive interventions are proposed without considering the development of recent novel technologies used to spread psychological interventions, such as Internet-based applications. 

### 1.1. The Use of the Internet to Improve Communication Skills: Advantages, Critical Issues, and Innovative Solutions

The Internet is the ultimate tool to develop and disseminate mental health interventions, as it provides a series of advantages: it can reach many people, even those that would not ask for help due to structural (e.g., high costs) and personal (e.g., fear of being stigmatized) barriers; it provides low-cost and always-accessible services for users, and it guarantees more privacy and anonymity [23,24]. Moreover, thanks to the interactive and dynamic nature of the Internet, it is possible to adjust content and graphics based on the target audience and to create online environments to ease the engagement of users [25], adding multimedia content [26,27]. A broad range of evidence supports the efficacy of Internet-based interventions, also called online or web-based intervention, in reducing symptoms related to common disorders [28,29,30,31], promoting positive mental health [32], satisfaction with life, self-esteem [33], and happiness [34], and in decreasing transdiagnostic factors [33,34,35,36,37,38]. 

Many studies included communication skills and assertiveness in more comprehensive online interventions, however in most of the studies, assertiveness is not evaluated as an outcome of the intervention, leaving the skill potential improvement unknown [39,40,41,42,43,44]. Studies that considered assertiveness as a primary outcome of the intervention reported inconsistent results, suggesting either no significant effect [36,45] or improvements in specific communication skills, but not in the general level of assertiveness [46]. 

The practice of assertive behavior is essential for the effectiveness of assertive training because it provides the opportunity to recreate a specific interpersonal situation, identify and change negative thinking patterns, evaluate irrational assumptions and expectations, decrease anxiety related to social situations [47], and enact all the appropriate behavioral responses [3]. At the same time, it is important to practice assertiveness in real-life situations, to train abilities, and to evaluate the consequences of assertive behavior [47]. This practical component of assertive training could be defective in Internet-based intervention, and it could explain the inconsistent findings of the current literature. The absence of human interactions that characterize Internet-based interventions do not guarantee supervised practice and a positive environment where the participant can interact adaptively with each other and the supervisor [48,49,50]. 

Therefore, a new approach must be found to guarantee adequate practice and foster the efficacy of Internet-based assertive interventions. We hypothesized that integrating imagery techniques within the most traditional assertive training could be an effective strategy [51]. Such a hypothesis resulted from a series of considerations. First of all, the extraordinary versatility of imagery-based techniques has been widely supported, and their effectiveness has been demonstrated both in clinical [52,53,54] and non-clinical practice [55,56]. Second of all, working with mental images provides several benefits: it allows one to experience events and their emotional consequences [57,58,59]; compared to verbal expression, mental imagery has a stronger power to evoke emotional states [60]; the neural mechanisms involved in imagination are comparable to those involved in actual perception, meaning that imagining is equivalent to act [61,62]; imagination is a behavioral and motivational booster, influencing behaviors and beliefs about the future [63]. Lastly, individuals often imagine conversing with others [64]. This specific form of imagery, called imagery interaction, allows one to experience different communication strategies, practice conversation before a real encounter, and can improve communication competence [65].

Therefore, within this background, we developed ComunicaBene: a guided Internet-based imagery training to promote assertiveness and reduce the negative emotions associated with social interactions. ComunicaBene has been designed for university students and developed on evidence-based approaches, such as Cognitive–Behavioral models, CBT [66], and the Nonviolent Communication model, NVC [67]. ComunicaBene has been developed on an e-learning platform, creating an interactive and dynamic website environment. ComunicaBene includes a series of online modules related to a specific topic (e.g., emotions, communication style, assertiveness). In particular, each module contains theoretical and practical content and multimedia materials to engage students’ attention [25]. 

### 1.2. Aim of the Present Study

The paper presents a study protocol for a randomized controlled trial to investigate the intervention’s efficacy and acceptability. In particular, we expect that ComunicaBene will effectively promote assertiveness and reduce negative emotions associated with social interactions. Moreover, a series of secondary outcomes will be considered. Especially when considering that assertiveness significantly predicts psychological well-being [68], we hypothesize a change in this dimension. Moreover, we hypothesize a change in emotional awareness, as nonassertive social functioning is significantly related to alexithymia [69]. Lastly, as worry and rumination are mostly verbal-based maladaptive emotional regulation strategies [70], we hypothesized that an increased use of mental imagery could reduce these thinking patterns [71]. The local Ethical Committee of the Department of Psychology, Sapienza University of Rome, approved the study protocol.

## 2. Materials and Methods

### 2.1. Study Design

**Figure 1:**
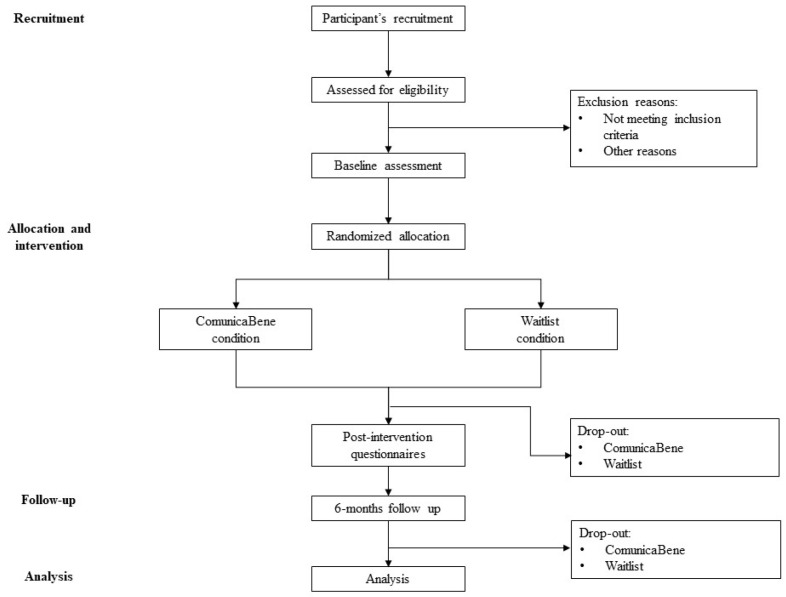
Design and participant flow chart.

A randomized control trial will be conducted, in which participants will be randomly allocated to an experimental or control condition. The ComunicaBene condition will consist of guided self-help imaginary training to improve assertiveness. The Waitlist, or control, condition will consist of a waiting list. Participants will receive the same intervention as those in the ComunicaBene condition but at a later time. Assessment will be run at baseline (pre-intervention assessment), at the end of the intervention (post-intervention assessment), and six months after the intervention (follow-up assessments). Please see Figure 1 for the design and participant flow chart.

### 2.2. Participants

Participants’ recruitment will be spread by word of mouth, mailing lists, and sharing fliers on various social networks (e.g., Instagram). Interested students will be invited to send an email to book their participation. ComunicaBene will be presented as imagery training to promote assertiveness, reduce the negative emotions and distress associated with interpersonal interactions, and to be able to build and maintain positive relationships. 

Eligible criteria will include enrollment in any degree course at the beginning of the study, having regular access to the Internet, the ability to write, hear, read, speak, and understand Italian, and an age between 18 and 35 years old. Exclusion criteria included the presence of a severe mental disorder (i.e., psychotic disorder, bipolar disorder) or the presence of suicidal ideation or risk.

#### Sample Size Calculation

The sample size has been estimated based on power analysis using G*Power 3.1. [72]. The following parameters were set on the sample size calculator: effect size of f = 0.21, mixed analysis of variance (ANOVA) including within-subject (3-time points), and between-subject (2 groups); effects were with 95% power and at a two-sided 5% significance level. Results suggested that the minimum required sample size was 60 participants. However, considering the percentage of drop-out occurring in Internet-based intervention for university students [73], a drop-out rate of around 25% was expected. For this reason, it is planned to recruit approximately 90 participants.

### 2.3. Measures

#### 2.3.1. Preselection Assessment

The Symptom Checklist-90-Revised, SCL-90-R [74,75], and the WHO Disability Assessment Schedule, WHODAS 2.0 [76,77] will be used to exclude the presence of a severe mental disorder or the presence of suicidal ideation or risk. The SCL-90-R is a self-report questionnaire that aims to evaluate a broad range of symptoms (i.e., somatization, obsessive compulsive, interpersonal sensitivity, depression, anxiety, hostility, phobic anxiety, paranoid ideation, and psychoticism). It also provides an index of overall psychological distress, of the intensity of symptoms, and the number of self-reported symptoms. Cronbach’s alpha for the Italian version ranges from 0.68 to 0.87. The WHODAS 2.0 is a 36-item questionnaire on a 5-point Likert scale. It allows evaluating the level of disability in six functioning domains: cognition (i.e., understanding and communicating), mobility (i.e., moving and getting around), self-care (i.e., hygiene, dressing, eating and staying alone), getting along (i.e., interacting with other people), life activities (i.e., domestic responsibilities, leisure, work, and school) and participation (i.e., joining in community activities). It provides scores for each dimension and a total score of disability. Considering the Italian version, at the domain level, reliability ranges from 0.93 to 0.96.

#### 2.3.2. Primary Outcome

Assertiveness. The Scale for Interpersonal Behavior-Short Form, s-SIB [78], will measure assertive behavior. It is a 50-item questionnaire divided into two main scales (25 items each): distress and performance, which measure the distress in state assertiveness and the probability of engaging in a specific assertive behavior, respectively. Moreover, each scale consists of five dimensions: negative assertion (i.e., requesting a change in others’ behavior, defending one’s rights and opinions, refusing requests), personal limitation (i.e., admitting one’s failures, faults, and shortcomings), initiating assertiveness (i.e., introducing oneself, starting conversations, expressing opinions), positive assertion (i.e., praising others, accepting compliments, displaying positive feelings), and general assertiveness. The Italian version showed good internal consistency with alpha coefficients ranging from 0.71 to 0.90 for the distress scale and 0.67 to 0.85 for the performance scale. 

#### 2.3.3. Secondary Outcome

Psychological well-being. The Psychological General Well-Being Index, PGWBI [79,80] will be used to evaluate subjective psychological well-being. It is a 22-item questionnaire on a 6-point Likert scale that produces a single measure of psychological well-being (total score) and a single score for six dimensions of well-being: anxiety, depression, positive well-being (i.e., satisfaction from working life and interest in the everyday activities), self-control (i.e., ability to regulates one’s emotions, behavior, desires, self-confidence and to take decisions), general health (i.e., perception of being in good health), and vitality (i.e., mental and physical fatigue, apathy, loss of energy). The PGWBI global score represents the sum of all items and ranges from 0 to 110, with higher scores indicating greater psychological well-being. Considering the Italian version, Cronbach’s alpha ranges from 0.77 to 0.88. 

Repetitive thinking. Repetitive thinking, such as rumination and worry, is a process of passive and uncontrollable thinking focused on negative content [70]. The Ruminative Response Scale, RRS [81,82], and the Anger Rumination Scale, ARS [83,84], will be used to measure depressive and anger rumination, respectively. The Ruminative Response scale is a 22-item questionnaire assessing the tendency to respond with rumination to depressed mood. The Cronbach’s alpha of the questionnaire is 0.90. The Anger Rumination Scale is a 19-item questionnaire that assesses the tendency to recall mind anger-provoking situations from the past and think about the causes and the consequences of anger episodes. In the Italian sample, the scale showed good internal consistency (α = 0.85). Moreover, the Penn State Worry Questionnaire, PSWQ [85,86], will be used to evaluate worry. It measures the tendency to worry, regardless of the specific content. The Cronbach’s alpha of the Italian version is 0.85. 

Emotional awareness. The Toronto Alexithymia Scale-20, TAS-20 [87,88] will be used to evaluate emotional awareness, namely, the ability to distinguish, recognize, and communicate emotions [89]. The TAS-20 is composed of three scales: difficulty describing feelings, difficulty identifying feelings, and the externally oriented thinking. It also provides a total score of alexithymia. The Cronbach’s alpha for the total score in the Italian version is 0.81. 

Imagined interaction. The Scale of Imagined Interaction, SII [90] evaluates the eight attributes (frequency, proactivity, retroactivity, discrepancy, variety, valence, self-dominance, and specificity) and the six functions (relational maintenance, compensation of real interaction, rehearsal, self-understanding, conflict, catharsis) of imagined interaction. The questionnaire begins with a brief explanation of what imagined interaction is; it comprises 60 items on a 7-point Likert scale, ranging from very strongly disagree to very strongly agree, and seven open questions about qualitative contents of the imagination. Considering the original version, Cronbach’s alpha ranges from 0.70 to 0.91 for the attributes dimensions and 0.71 to 0.90 for the frequency dimensions. The scale has been translated and back-translated into Italian. 

#### 2.3.4. Other Measures

Sociodemographic Questionnaire. An ad-hoc questionnaire will be used to collect information about the residence, professional status, psychological past/current treatment, and educational level.

Feedback questionnaire. An ad-hoc questionnaire will be proposed to collect feedback and suggestions from participants in the ComunicaBene condition. It includes 26 items, with five open questions and 21 on a 5-point Likert scale. The questionnaire aims to collect information about the experiences with the Tutor, the general utility of the training, the acceptability, and the feasibility of the contents and activities. Please see Section S1 in the Supplementary Materials for the entire questionnaire.

Treatment fidelity questionnaire. After every meeting, the Tutor will have to complete an ad-hoc questionnaire to ensure the treatment fidelity was guaranteed [91]. The questionnaire is composed of 10 questions: 7 open questions (e.g., “issues discussed during the meeting”, “difficulties reported by the students”); 1 yes or no question (i.e., “the student was in touch with you between the meetings?”); and 2 multiple choice questions (i.e., “module discussed during the meeting”, “duration of the meeting”). 

### 2.4. Procedure

Every participant will first sign the institutional consent forms, including the study’s goals, procedure, risks, and data protection and privacy information according to the General Data Protection Regulation (EU) 2016/679 (GDPR). Once achieved, each participant will receive a username and password to log in to their private page on the website. Participants will find a first assessing module containing the sociodemographic questionnaire and the SCL-90-R and WHODAS 2.0 questionnaires to detect clinical disorders. Based on the exclusion criteria, in the absence of any significant clinical condition, participants will be randomly assigned to either the ComunicaBene or Waitlist condition. Before, at the end, and 6 months after the end of the intervention, participants will complete a series of standardized questionnaires to assess assertiveness (SIB-S), psychological well-being (PGWBI), depressive rumination (RRS), anger rumination (ARQ), worry (PSWQ), alexithymia (TAS-20), and imagined interaction (SII). Moreover, an ad-hoc questionnaire will also be administered at the end of the intervention to evaluate satisfaction with the program. Every participant will find the questionnaires on their private page on the website.

#### 2.4.1. ComunicaBene Condition 

Participants in this condition will use their username and password to log in to their private page on the website. ComunicaBene provides different online training modules that are presented in three consecutive phases. Below, an outline for each step is presented. 

(1) Psychoeducation and motivation. This phase aims to help students recognize and distinguish their own and others’ communication styles (assertive, passive, or aggressive), understand and learn the principles of assertive communication, and develop awareness about their own needs and emotions, especially the one that can be an obstacle to communicate assertively [3]. At first, participants will be encouraged to set specific goals (e.g., “I would like to improve the ability to say no to my parents”; “I would like to be able to express my disagreement to my Supervisor”) that they want to achieve with the program, focusing on the most challenging interpersonal situations, such as the factors that hinder assertive communication, and the cost and benefits of changing. Later, they will find theoretical and practical content about needs, emotions, communication style, and assertiveness. Also, they will have to report their significant interaction in a personal diary [1]. Moreover, this phase also includes a preparation session to allow students to train their imaginative skills. The participant will be asked to recall a specific, concrete, and real memory or to imagine future actions [51]. 

(2) Imagery sessions. Participants will find thirty audio tracks that have been developed ad hoc, based on typical interpersonal problems of the target population (an example of audio track is available under request to the corresponding author). Since assertiveness is a situation-specific behavior and can be affected by personal, situational, and interactional influences [92], every track is content-interlocutor-specific. In particular, there are six possible situations (express disagreement, decline a request, make a request, express constructive criticism, handle criticism, and express positive emotions) combined with five interlocutors (partner, friends, familiar, colleagues, and superior). Every track will be listened to twice, starting with the easiest situation [52]. Each audio track includes a guided imagery technique in which a voice guides individuals through evoking specific interpersonal situations. This technique, based on process and end-state imagery [93], helps participants in focusing on the necessary steps for assertive communication (process imagery) and visualizing the desired outcome (end-state imagery). In particular, every audio track is composed of eight phases: (1) pre-relaxation phase, (2) introduction of the situation and questions to create vivid and realistic mental imagery, (3) imagination of their spontaneous response with associated consequences, (4) instruction for the assertive response, (5) repetition of the assertive response with the guide, (6) repetition of the assertive response without the guide, (7) focus on the consequences of assertiveness, and (8) conclusion. Every audio tracks last about 10 min. After every listening, students will complete an ad-hoc journal to stimulate a post-audio track reflection [1]. The journal includes questions to identify feelings experienced before, during, and after the imaginative session, investigate the degree of satisfaction with the session, and identify the negative thoughts haven during the session. Students are especially helped to identify cognitive biases, irrational thoughts, and catastrophic scenarios. Lately, students have been helped to replace negative thoughts and scenarios with alternative and more realistic ones [94]. 

(3) Learning generalization. This phase aims to ensure learning generalization by asking students to present what they learned during imaginative practice sessions in real-life situations. Students will have to monitor their significant interactions using a self-monitoring tool.

**Figure 2:**
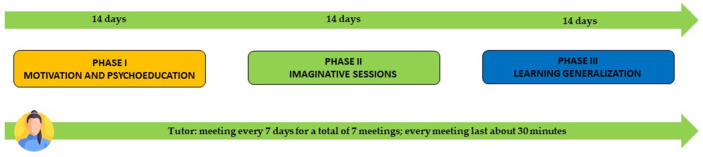
Timing ComunicaBene protocol.

The program includes periodical supervision via a Tutor. The Tutors will be psychologists previously trained in delivering the intervention, using the platform, and becoming familiar with CBT and NVC models. In particular, the Tutor will have to maintain and stimulate motivation and commitment, monitor the student’s progress on the website, and set a video-call meeting every week with their students. During the online meeting, Tutors and students will review the activities, discuss the most critical content, explain unclear concepts, and address any difficulties encountered. Moreover, the Tutor will have a modeling role: it can interpret the communicator and serve as a model of assertive behavior [95]. Lastly, the Tutor will have to realistically reinforce participants’ effort and success and constructively criticize dysfunctional behaviors [96]. Every Tutor will have to adhere to an ad-hoc protocol created for the intervention to guarantee treatment fidelity (Please see Section S2 in the Supplementary Materials for the entire protocol). Moreover, Tutors will be periodically supervised by an expert psychotherapist who is also the creator of the intervention. Please refer to Figure 2 for a timeline of the intervention.

#### 2.4.2. Waitlist Condition

Participants in this condition will not receive training but return for the post-intervention and follow-up assessment. After the study ends, participants will be invited to participate in the intervention.

## 3. Statistical Analysis

SPSS (IBM) version 27 will be used to process data. Descriptive analyses will be run on sociodemographic variables, data related to the satisfaction questionnaires, and SCL-90-R and WHODAS 2.0 scores. Chi-Square tests and Student’s *t*-tests will be run to investigate differences between participants in the two conditions on baseline demographic variables, gender variables, age, and all baseline measures. If some variables significantly differ between conditions, they will be considered as covariants in the following analyses. A series of mixed models of analysis of variance (ANOVAs) will be run to investigate the efficacy of the intervention on the SIB-S, PGWBI, RRS, ARQ, PSWQ, TAS-20, and SII scores, as a function of the factor between CONDITION (ComunicaBene vs. Waitlist) and the factor within TIME (Baseline vs. Post-Intervention vs. Follow-Up). The level of significance will be set at *p* < 0.05. Effect sizes will be calculated using partial eta squared and interpreted based on benchmarks that Cohen (2013) suggested: η_p_^2^ = 0.01, small effect size; η_p_^2^ = 0.06, medium effect size; η_p_^2^ = 0.14 large effect size. The post hoc analyses of significant interaction will be conducted using the LSD post hoc test.

## 4. Discussion

The importance of communication skills for mental health and self-realization is widely accepted [4,5,6,7]. However, despite its clear usefulness, research on assertiveness and assertive training has declined significantly in the past decades [10]. Consequently, traditional training does not consider the most recent novel technologies used to spread psychological interventions. This is not in line with the more recent evidence suggesting the importance of using innovative strategies to deliver mental health interventions, such as the Internet, to reach more people, reduce costs, and overcome structural barriers [24]. Overall, while promoting assertiveness through the Internet offers many benefits, some potential obstacles and criticisms need to be considered. For example, online assertive training may not provide the same level of personal interaction as in-person training, and it can compromise the needed practice required in assertive training. Moreover, online training may not provide the same feedback and support as in-person training. This can make it more difficult for individuals to assess their progress and improve their assertiveness skills. 

ComunicaBene, a guided Internet-based imagery intervention to promote assertiveness, has been developed to address these challenges. This paper presents the study protocol for a randomized controlled trial that will be conducted to investigate the efficacy and the acceptability of the intervention. 

ComunicaBene includes some of the phases of traditional assertive training: the initial assessment, which aims to evaluate the level of assertiveness and to identify the most difficult interpersonal situations; the commitment and motivation phase, which aims to help participants consider the costs and benefits of changing and to set personal goals; and the psychoeducational phase, which aims to help participants to recognize and distinguish different communication styles, to learn the main principles of assertive communication, and to improve their awareness and to accept their rights and others’ rights. We hypothesize that working on these elements will help participants become more aware of their needs, emotions, communication style, and interpersonal functioning [3].

The main difference corresponding to the innovative strategies proposed with ComunicaBene is the substitution of the in vivo-practice with imagery practice. Practice is an essential component of every assertive training, and, as previously mentioned, it can be insufficient or absent in online assertive training. The use of imagination could fill this gap, as mental imagery allows one to create, reactivate, and manipulate internal representations without a direct external stimulus [62]. We hypothesized that by mentally rehearsing specific behaviors and scenarios, participants would enhance their communication skills and improve their overall social performance [51]. Specifically, we believe that allowing individuals to create a positive mental image of the future and visualizing themselves performing at their best will increase their confidence and self-efficacy [56,97,98,99]. Moreover, by mentally rehearsing specific interpersonal scenarios, individuals can prepare themselves mentally for potential challenges, reducing uncertainty and distress [90,100]. Overall, we hypothesized that behavior rehearsal and in vivo-role playing could be replaced with imagery techniques, making assertiveness practice possible via the Internet. 

ComunicaBene will also include an in vivo-exposure, as participants in the last phase will have to act out what they have previously learned in real-life situations. The aim is to improve and generalize their learning by practicing their ability and evaluating their change and the consequences of assertive behavior [3]. Therefore, we hypothesize that even after the end of the training, participants will be able to act assertively even in entreated situations [47] and that thanks to repeated practice, the improvements could be maintained over time [1].

Moreover, as previously mentioned, ComunicaBene includes regular monitoring of a Tutor. Therefore, we expect adherence to the intervention, as the Tutor helps participants to maintain motivation in carrying out the program [101]. Moreover, we believe that the Tutors may foster the effectiveness of the intervention and promote change in the participants, as they will serve as a model of assertive behavior [95], a reinforcement of students’ effort and success [96], and will help participants to identify cognitive biases, irrational thoughts, and catastrophic scenarios, and replace them with alternative and more realistic ones [94]. 

Regarding the acceptability of the intervention, we expect that participants will be satisfied with the program, as young people usually report high satisfaction and perceived utility with online programs [23]. 

Besides these expectations, it is also important to consider some possible limitations and obstacles: ComunicaBene is a quite brief intervention, and changes may occur after more in vivo exposure [102]. Moreover, the situation proposed in the audio tracks may not fit the needs of every participant. Participants’ difficulty immersing themselves in the imagined interactions proposed could compromise the effectiveness of the imaginative sessions. If this study will point out this limitation, we will develop more audio tracks, allowing students to choose the one that better suits their needs and experiences. 

In conclusion, we expect some noteworthy results from this study. First of all, the study’s findings will shed light on the potential effect of ComunicaBene on assertiveness. Considering the role of assertiveness in contributing to mental health and well-being [4,5] and the role of unassertiveness in contributing to psychopathology [10], ComunicaBene may be both an efficacious prevention and promotion intervention. Moreover, it is a scalable and affordable intervention and could overcome all the limitations related to traditional services, such as high costs, lack of services in specific areas, and long waiting lists [24]. ComunicaBene has been designed for university students; however, the program can easily be adapted to other target audiences (e.g., workers, parents, and teachers) by developing new audio tracks based on their specific needs and characteristics. Therefore, the applicability of ComunicaBene could even be expanded. Moreover, this study will propose a new insight into integrating imagery techniques within assertiveness training. Lastly, the findings of this study will provide new evidence about the use of the Internet to spread communication skills training. It will provide a series of advantages: individuals can easily access anytime and anywhere to reliable resources, have the opportunity to practice assertiveness with a Tutor at a reduced cost, compared to in-person training, and the Tutor can constantly monitor the participants’ progress, providing them feedback and reinforcement; moreover, to conclude, interventions spread through the Internet can reach a great number of people.

## Data Availability

Not applicable.

## Supplementary Materials

The following supporting information can be downloaded at: https://www.mdpi.com/article/10.3390/healthcare11131874/s1, Section S1: Feedback questionnaire; Section S2: Tutor protocol.
