# Supplementary material for: Imagine All the People: A Guided Internet-Based Imagery Training to Increase Assertiveness among University Students—Study Protocol for a Randomized Controlled Trial
Source: Healthcare (Basel). 2023 Jun 28;11(13):1874. doi: 10.3390/healthcare11131874 (PMC10341122; doi:10.3390/healthcare11131874)
Supplement: Supplementary file 1 [file healthcare-11-01874-s001.zip › Section S1.pdf]

Section S1. Feedback questionnaire

We ask you to answer the following questions concerning your experience with ComunicaBene. Please remember that there is no right or wrong answer. Your opinion is essential to improve the program.

Please consider the following scale when answering the questions.

1<br>Strongly disagree	2<br>Disagree	3<br>Neither agree nor disagree	4<br>Agree	5<br>Strongly agree

ComunicaBene helped me to improve my well-being	1	2	3	4	5
ComunicaBene helped me to know myself better	1	2	3	4	5
ComunicaBene helped me to improve myself	1	2	3	4	5
ComunicaBene helped me to improve my communication skills	1	2	3	4	5
ComunicaBene helped me to improve the quality of my relationships	1	2	3	4	5

Now please fill out the following open questions

Would you suggest ComunicaBene to other students? If not, why?

Any feedback and suggestions

We ask you to answer the following questions concerning your experience with the Tutor.

Please consider the following scale when answering the questions.

1<br>Strongly disagree	2<br>Disagree	3<br>Neither agree nor disagree	4<br>Agree	5<br>Strongly agree

I considered useful the presence of the Tutor	1	2	3	4	5
The Tutor helped me to understand misunderstanding contents	1	2	3	4	5
The Tutor helped me to understand my feelings and thoughts better	1	2	3	4	5
The Tutor helped me to improve my communication skills	1	2	3	4	5
The Tutor helped me to do not to lose my motivation	1	2	3	4	5

Any feedback or opinions about the Tutor					
We ask you to answer the following questions concerning the modules you completed.<br><br>Please consider the following scale when answering the questions.<br>1 Strongly disagree / 2 Disagree / 3 Neither agree nor disagree / 4 Agree / 5 Strongly agree					
Phase 1. Psychoeducation and motivation					
The instructions to set personal goals were clear and understandable	1	2	3	4	5
The theoretical contents about emotions were understandable	1	2	3	4	5
The theoretical contents about communicative style were understandable	1	2	3	4	5
The exercises about emotions were useful for understanding the components of emotions	1	2	3	4	5
The exercises about assertiveness were useful for understanding the pillars of assertiveness	1	2	3	4	5
The self-monitoring was useful for helping me understand my feelings	1	2	3	4	5
The self-monitoring was useful for helping me understand my communicative style	1	2	3	4	5
Phase 2. Imagery sessions					
I appreciated the audio tracks	1	2	3	4	5
Now please fill out the following open question					
What do you think about the duration of the audio tracks?					
Any feedback or opinions about the Tutor					
Phase 3. Learning generalization					
The self-monitoring was useful for helping me understand my feelings	1	2	3	4	5

The self-monitoring was useful for helping me understand my communicative style	1	2	3	4	5
I carried out in real-life situations what I learned in the imagery sessions.	1	2	3	4	5
