# Supplementary material for: Imagine All the People: A Guided Internet-Based Imagery Training to Increase Assertiveness among University Students—Study Protocol for a Randomized Controlled Trial
Source: Healthcare (Basel). 2023 Jun 28;11(13):1874. doi: 10.3390/healthcare11131874 (PMC10341122; doi:10.3390/healthcare11131874)
Supplement: Supplementary file 1 [file healthcare-11-01874-s001.zip › Section S2.pdf]

Section S2. Tutor protocol

Meeting	Goal, activities, and homework
1st meeting	Mutual introduction, presentation of the program, and tutoring organization.<br>Review of the "My Motivation to Change" module:<br>● Did the student set goals in line with ComunicaBene's contents?<br>● Are the goals realistic, measurable, specific, and reachable?<br>● Did the student identify the obstacle to assertiveness?<br>Discuss any encountered difficulties and possible future difficulties.<br>Homework: Emotions, assertiveness, and training to imagination module.
2nd and 3rd meeting	Review of the Emotions, assertiveness, and training to Imagination module<br>● Did the students understand the ABC model of emotions?<br>● Discuss some emotional episodes following the ABC model<br>● Discuss students' communication styles with examples of real interactions<br>Review the interaction self-monitoring diary.<br>● Did the student consider this tool useful?<br>● Did the students encounter any difficulties/feel distressed in completing the diary?<br>● Discuss the social interaction reported in the diary, focusing on the obstacle in being assertive and on the factors that otherwise facilitate assertive communication<br>Did the students listen to the preparatory audio track?<br>● Did the student consider this exercise useful?<br>● Did the students encounter any difficulties/feel distressed in listening to the track?<br>Homework: Imaginative session module

4th meeting	Review of the Imaginative session module<br>● How many audio-track the students listen to?<br>● Did he respect the order of the audio track?<br>● Did the students encounter any difficulties/feel distressed in listening to the track?<br>Discuss the emotions and thoughts experienced during the audio track, focusing on the obstacles of being assertive<br>● For every obstacle identified, even though the post-track journal helps students to formulate alternative thoughts and decatastrophize the identified negative consequences.<br>Homework: Imaginative session module
5th meeting	Review of the Imaginative session module<br>● How many audio-track the students listen to?<br>● Did he respect the order of the audio track?<br>● Did the students encounter any difficulties/feel distressed in listening to the track?<br>Discuss the emotions and thoughts experienced during the audio track, focusing on the obstacle of being assertive<br>● For every obstacle identified, even though the post-track journal helps students to formulate alternative thoughts and to catastrophize the negative consequences identified.<br>Homework: Learning generalization module
6th meeting	Review of the Learning generalization module<br>Review the interaction self-monitoring diary.<br>● Did the student consider this tool useful?<br>● Did the students encounter any difficulties/feel distressed in completing the diary?<br>● Discuss the social interaction reported in the diary, focusing on the obstacle in being assertive and on the factors that otherwise facilitate assertive communication<br>Homework: Learning generalization module

7th meeting	Review of the Learning generalization module<br>Review the interaction self-monitoring diary.<br>● Did the student consider this tool useful?<br>● Did the students encounter any difficulties/feel distressed in completing the diary?<br>● Discuss the social interaction reported in the diary, focusing on the obstacle in being assertive and on the factors that otherwise facilitate assertive communication<br>Final feedback<br>● Which goals have been achieved? Which not?<br>● Which obstacles hindered the achievement of the goals?<br>● Which new competencies and resources have the students now to achieve these goals?
